# First person – Nayeli Reyes-Nava

**DOI:** 10.1242/dmm.052746

**Published:** 2025-12-29

## Abstract

First Person is a series of interviews with the first authors of a selection of papers published in Disease Models & Mechanisms, helping researchers promote themselves alongside their papers. Nayeli Reyes-Nava is first author on ‘ Physical and functional interaction of the ciliopathy proteins Lrrc56 and Odad3 control deployment of axonemal dyneins in vertebrate multiciliated cells’, published in DMM. Nayeli is a postdoc in the lab of John Wallingford and Edward Marcotte at the University of Texas at Austin, Austin, TX, USA, investigating how large ciliary protein complexes are trafficked into cilia to reveal the molecular basis of ciliopathies.


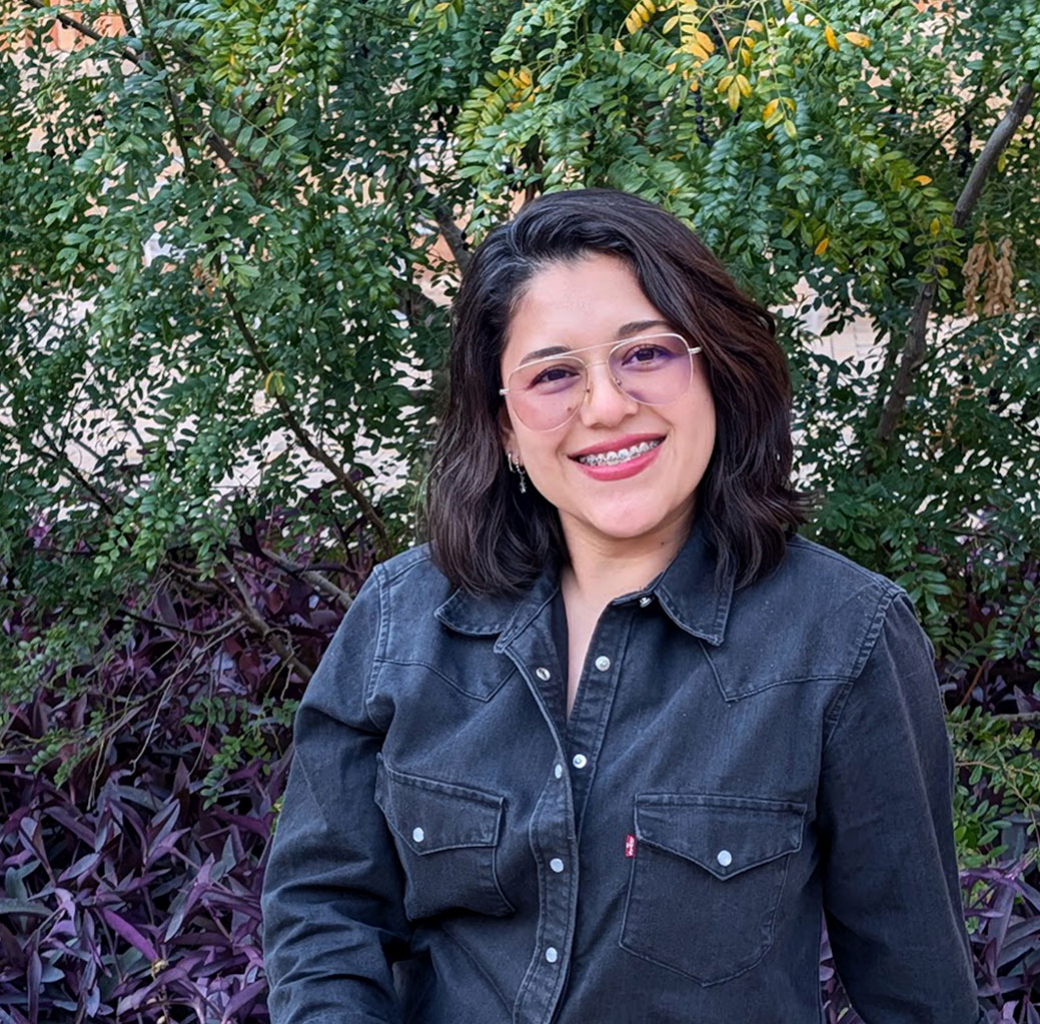


**Nayeli Reyes-Nava**

**Who or what inspired you to become a scientist?**

My passion for science took root studying plant biology as an undergrad, inspired by the time I spent in my grandmother's garden as a kid. Eventually, that spark grew into a lifelong passion for discovery. Science kind of found me.

**What is the main question or challenge in disease biology you are addressing in this paper? How did you go about investigating your question or challenge?**

I care deeply about rare disorders – it still amazes me how common they truly are, yet how little we understand about their underlying biology. My research aims to close part of this gap by dissecting the cellular and molecular mechanisms that drive motile ciliopathies, inherited disorders that can lead to infertility, hydrocephalus and chronic lung disease due to non-functional motile cilia – hairlike organelles essential for tissue homeostasis and cell movement.

My main question is how large ciliary protein complexes essential for cilia motility, such as outer dynein arms (ODAs) and the ODA docking complex, are assembled and trafficked in the cytosol of multiciliated cells. To investigate this, we used *Xenopus* live imaging, loss-of-function approaches and proteomics to define the molecular machinery that underlies motile cilia function and how its disruption contributes to ciliopathies.

**How would you explain the main findings of your paper to non-scientific family and friends?**

Some cells in our bodies have dozens of tiny, hair-like structures on their surface called cilia. These cilia beat together like little oars to keep things moving – clearing mucus and germs from our lungs, helping fluid move inside the brain and even helping with reproduction. When these cilia don't work properly, people can develop serious health problems, including repeated lung infections that can be so severe they may eventually require a lung transplant.

In our study, we wanted to understand how these cilia are built. In particular, we focused on how large ‘motor’ structures – proteins that make cilia beat – are moved from where they are made inside the cell to the cilia at the surface. We studied a protein called LRRC56, because people with changes (mutations) in the encoding gene often develop severe lung disease. Earlier research also suggested that Lrrc56 helps move important cilia-building parts to where they need to go.

What we found is that Lrrc56 works closely with other proteins that help anchor the cilia's motors in place. When Lrrc56 is changed – even in just one part of the protein – it can't go to the right place in the cell, and the motors can't be properly attached. We also showed that mutations found in patients prevent Lrrc56 from interacting normally with these partner proteins.

In simple terms, our work shows how a single damaged protein can disrupt the ‘assembly line’ that builds working cilia, helping explain why some people develop severe lung disease.

… our work shows how a single damaged protein can disrupt the ‘assembly line’ that builds working cilia, helping explain why some people develop severe lung disease

**What are the potential implications of these results for disease biology and the possible impact on patients?**

Motile ciliopathies don't have a cure, so understanding the biology behind these disorders is really important. Figuring out which components help move essential cilia-building proteins inside the cell is not only fascinating from a basic science perspective, but also informative for patients and potentially helpful for diagnosis. In our work, we found that a single trafficking protein may coordinate the transport of both the ciliary motors and the docking machinery that anchors them. Understanding how these processes work is a key step toward developing more targeted and personalized treatments in the future.

**Figure:**
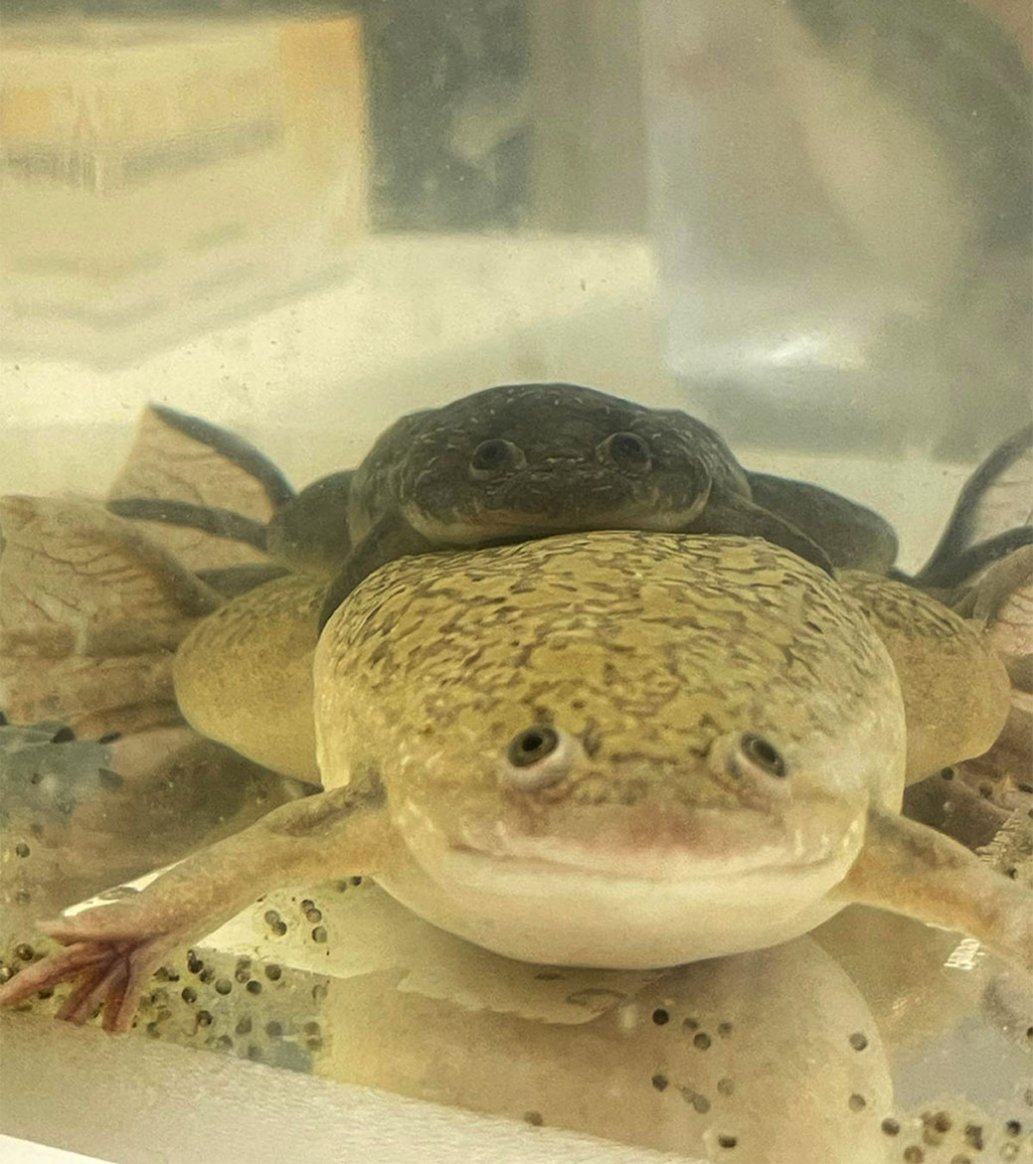
A mating pair of *Xenopus* frogs with newly laid eggs – embryos we use as a model for studying cilia biology.

**Why did you choose DMM for your paper?**

It might sound cliché, but submitting to DMM was a grad school dream of mine. When Dr Wallingford asked where I wanted to send this paper, I didn't hesitate. Mission accomplished!

**Given your current role, what challenges do you face and what changes could improve the professional lives of other scientists in this role?**

One of the biggest challenges in my current role is the uncertainty in the job market and the impact of funding cuts. These days, postdocs can't just focus on being postdocs – we have to plan our futures in the middle of this uncertainty. That pressure makes it harder to focus solely on research, even though that's the core of what we do. What helps tremendously are strong research programs and supportive communities; having colleagues and mentors to share experiences with, learn from and lean on makes a huge difference in navigating this stage of our careers.

**What's next for you?**

I'm fortunate to be at a place with incredible resources and a supportive community, where I'll continue my postdoctoral training for the next couple of years. Beyond that, my goal is to establish my own independent research lab, where I can continue investigate the cellular and molecular mechanisms underlying disease and train the next generation of scientists.

**Tell us something interesting about yourself that wouldn't be on your CV**

I've worked in several coffee shops, so I'm a trained barista, and I love specialty coffee. Making the perfect cup takes patience and precision – skills that come in handy in the lab too!
